# Chaotic Enhanced Genetic Algorithm for Solving the Nonlinear System of Equations

**DOI:** 10.1155/2022/1376479

**Published:** 2022-04-12

**Authors:** A. M. Algelany, M. A. El-Shorbagy

**Affiliations:** ^1^Department of Mathematics, College of Science and Humanities in Al-Kharj, Prince Sattam Bin Abdulaziz University, Al-Kharj 11942, Saudi Arabia; ^2^Department of Mathematics, Faculty of Sciences, Fayoum University, Fayoum 63514, Egypt; ^3^Department of Basic Engineering Science, Faculty of Engineering, Menoufia University, Shebin El-Kom 32511, Egypt

## Abstract

Many engineering and scientific models are based on the nonlinear system of equations (NSEs), and their effective solution is critical for development in these domains. NSEs can be modeled as an optimization problem. So, the goal of this paper is to propose an optimization method, to solve the NSEs, which is called a chaotic enhanced genetic algorithm (CEGA). CEGA is a chaotic noise-based genetic algorithm (GA) that improves performance. CEGA will be configured so that it uses a new definition which is chaotic noise to overcome the drawbacks of optimization methods such as lack of diversity of solutions, the imbalance between exploitation and exploration, and slow convergence of the best solution. The goal of chaotic noise is to reduce the number of repeated solutions and iterations to speed up the convergence rate. In the chaotic noise, the chaotic logistic map is utilized since it has been used by numerous researchers and has proven its efficiency in increasing the quality of solutions and providing the best performance. CEGA is tested using many well-known NSEs. The suggested algorithm's results are compared to the original GA to prove the importance of the modifications introduced in CEGA. Promising results were obtained, where CEGA's average percentage of improvement was about 75.99, indicating that it is quite effective in solving NSEs. Finally, comparing CEGA's results with previous studies, statistical analysis by Friedman and Wilcoxon's tests demonstrated its superiority and ability to solve this kind of problem.

## 1. Introduction

Many models in engineering and science are based on the nonlinear system of equations (NSEs), and their solution is very critical for development in these fields. NSEs can be found directly in some applications, but they can also be found indirectly when practical models are transformed into NSEs [1]. Finding a robust and effective solution for the NSEs might be a difficult task in theory.

The bisection technique, Muller's method, false-position method, Levenberg–Marquardt algorithm, Broyden method, steepest descent methods, branch and prune approach, Halley's method, Newton/damped Newton methods, and Secant method have traditionally been used to solve NSEs [2]. Secant and Newton are the methods of choice for solving NSEs in general. Some techniques, on the other hand, turn the NSEs into an optimization problem [3], which is subsequently solved using the augmented Lagrangian method [4]. These approaches are time-consuming, may diverge, are inefficient when solving a set of nonlinear equations, require a tedious process to calculate partial derivatives to build the Jacobian matrix, and are sensitive to initial conditions [5].

Because of these constraints, the researchers used evolutionary algorithms (EAs) to solve NSEs. EAs are a sort of metaheuristic that is often used to address problems of optimization that are too difficult to solve using traditional methods. EAs such as the genetic algorithm (GA) [6–8], particle swarm algorithm (PSO) [9, 10], artificial bee colony (ABC) [11], cuckoo search algorithm (CSA) [12], and firefly algorithm (FA) [13] have been used to solve NSEs. In [6], Chang proposed a real-coded GA for solving the nonlinear system. In [7], Grosan and Abraham offered a novel approach based on GA for dealing with the problem of complex NSEs by recasting it as a multiobjective optimization problem. In [8], an efficient GA with symmetric and harmonic individuals was used to solve NSEs. Mo et al. in [9] presented a conjugate direction to PSO for addressing NSEs, which merges the conjugate direction method (CDM) into PSO to enhance it and enable for fast optimization of high-dimensional optimization problems. By moving the challenge of high-dimensional function optimization to low-dimensional, CDM aids PSO in avoiding local minima. Jaberipour et al. suggested a new version of PSO for solving NSEs, which is based on a novel way of updating each particle's location and velocity [10]. To tackle the drawbacks of the classic PSO approach, such as trapping in local minima and delaying convergence, they changed the way each particle was updated. Also, Jia and He presented a hybrid ABC technique for solving NSEs in [11], which combined the ABC and PSO algorithms. The hybrid algorithm corrects the problem of sinking into a premature or local optimum by integrating the benefits of both strategies. Furthermore, in [12], Zhou and Li proposed an upgraded CSA to handle the NSEs. They employed a novel encoding strategy that ensures the provided solution is achievable without requiring the cuckoo's evolution to be altered. Finally, in [13], enhanced FA to solve NSEs as an optimization problem is introduced by Ariyaratne et al. with several advantages such as eliminating the need for beginning assumptions, differentiation, or even function continuity and allowing it to provide many root estimates at the same time.

The genetic algorithm (GA), based on natural selection, genetics, and evolution, was presented in 1975 [14] and described in 1989 [15] as a competent global strategy for tackling optimization problems. GA is well suited to solving optimization issues, and it continues to pique academics' interest. According to the literature, GA was commonly used to solve NSEs, where Mangla et al., in [16], highlight flaws in existing approaches (Bisection, Regula Falsi, Newton–Raphson, Secant, Muller, and so on) and justify the GA's application to NSEs while an approach for sorting out NSEs to solve them using the fixed-point method was proposed in [17], with the equations' arrangement determined by a GA that works with a population of the possible resolution procedures for the system. In addition, in [18], Ji et al. presented an optimization approach based on clustering evolution for obtaining an optimum piecewise linear approximation of a set of nonlinear functions. The technique is built on a balance of approximation precision and simplicity, and it enhances the approximate linear with the fewest possible departments. In [19], a GA technique to solve NSEs for a variety of applications is presented, in which the roots of NSEs were approximated using population size, degree of mutation, crossover rate, and coefficient size. Also, a method for solving nonlinear equations using GA was given in [20]. Furthermore, in [21], evolutionary algorithms to solve NSEs were used, which were turned into an unconstrained optimization problem with some basic mathematical relations. Finally, in [22], a new intelligent computer strategy for solving nonlinear equations based on evolutionary computational approaches was proposed mainly based on variants of GAs. But, when it works with complex and massive systems, however, GA has some downsides, including being extremely slow and making it hard to identify the global optimal solution due to the increased number of iterations required or long search time.

From this motivation, this study offers an algorithm that solves one of the most significant drawbacks with GA and all EAs which is the repeating of solutions during the optimization process, which wastes time. The proposed optimization algorithm is called a chaotic enhanced genetic algorithm (CEGA). Chaotic is a mathematical strategy that has been shown to improve the performance of numerous optimization algorithms. It has received a great deal of attention, and it has been applied in a range of domains including optimization [23]. The proposed CEGA is a combination between GA and chaotic noise. The chaotic noise is used when the solutions are repeated, during the optimization process of GA, to change the positions of the solutions chaotically. This combination aims to enhance GA by overcoming its drawbacks such as lack of diversity of solutions, the imbalance between exploitation and exploration, and slow convergence of the best solution.

The major contributions of this paper include the following:

1. Proposing a new methodology called a chaotic enhanced genetic algorithm (CEGA) to solve NSEs by using a combination between GA and chaotic noise
2. Presenting sufficient diversity of the solutions, and preventing consuming time during the optimization process by overcoming repetition of solutions
3. Ensuring improvement in every iteration by using chaotic noise and fast convergence to best solutions
4. Testing CEGA by many well-known NSEs
5. Using statistical tests to determine the relevance of the CEGA findings
6. Showing that CEGA is competitive and better than other optimization algorithms

The following is how the paper is structured.  Section 2 discusses nonlinear systems of equations. The proposed technique is detailed in Section 3. The numerical findings and discussions are shown in Sections 4 and 5, respectively.  Section 6 concludes with observations and conclusions.

## 2. Nonlinear System of Equations

The mathematical definition of a nonlinear system of equations (NSEs) is

(1) $$\begin{array}{c} \text{SNLE}=\left\{\begin{array}{c} f_{1}\left(z\right)=0,\\ f_{2}\left(z\right)=0,\\ \vdots \\ f_{Q}\left(z\right)=0, \end{array}\right.\; \end{array}$$

where  *z*=(*z*_1_, *z*_2_,…, *z*_*n*_) is a vector of *n* components subset of ℝ^*n*^, and *f*_*q*_∀ *q*=1,2,…, *Q* are the nonlinear functions that translate the *n*-dimensional space ℝ^n^'s vector *z*=(*z*_1_, *z*_2_,…, *z*_*n*_) to the real line. Some of the functions may be nonlinear, while others are linear. Finding a solution for NSEs entails finding a solution in which each of the *Q* functions above equals zero [24].

**Definition 1 .**

If ∀*q*=1, ..., *Q*, the functions *f*_*q*_(*z*)=0, then the solution *z*=(*z*_1_^*∗*^, *z*_2_^*∗*^,…, *z*_*n*_^*∗*^) is called the optimal solution of the NSEs.

Many approaches [25–27] transform the NSEs into an unconstrained optimization problem by the inclusion of the left side of all equations and the use of the absolute value function as

(2) $$\begin{array}{c} \begin{array}{c} F\left(z\right)=\text{abs}\left(f_{1}\left(z\right)+f_{2}\left(z\right)+\cdots +f_{Q}\left(z\right)\right),\\ \text{subject to }\left\{\begin{array}{c} f_{1}\left(z\right)=0,\\ f_{2}\left(z\right)=0,\\ \vdots \\ f_{Q}\left(z\right)=0, \end{array}\right. \end{array} \end{array}$$

where *F*(*z*) denotes the objective function. If all of the nonlinear equations are equal to zero (*f*_*q*_=0 ∀ *q*=1, ..., *Q*), the objective function in (2) has a global minimum.

## 3. The Proposed Methodology

This section provides an overview of GA and chaos theory. The suggested CEGA is next presented in detail.

### 3.1. Genetic Algorithm

**Figure 1:**
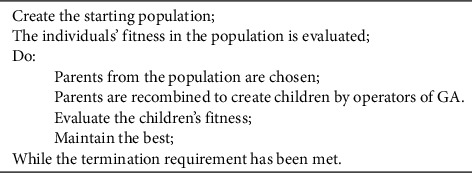
The pseudocode of the general GA.

In 1975 and 1989, respectively, Holland and Goldberg proposed and defined the genetic algorithm (GA) as an optimization technique [14, 15]. GA begins with a collection of chromosomes (solutions). Then, using GA operators (selection, mutation, and crossover), a new set of chromosomes is generated (solutions). The freshly generated chromosomes will be of greater quality than the preceding generation. These procedures are repeated until the termination conditions are met. As a final solution, the best chromosome (solution) of the previous generation is offered.  Figure 1 depicts the generic GA's pseudocode.

### 3.2. Chaos Theory

Chaos theory is concerned with the behavior of systems that obey deterministic laws yet look random and unpredictable. Many elements of the optimization sciences have benefited from the mathematics of chaos theory. Chaos optimization algorithms have received a lot of attention as a novel method of global optimization because they are based on many chaotic maps, and the inherent characteristics of chaotic maps can improve optimization algorithms by allowing them to escape from local solutions and increase the convergence to reach the global solution. To increase solution quality, many researchers advocated integrating chaos theory and optimization algorithms [28–31]. Chaotic maps are maps (evolution functions) that display chaotic behavior and typically take the form of iterated functions. Many well-known chaotic maps may be found in the literature, including the sinusoidal map, Chebyshev map, singer map, tent map, sine map, circle map, Gauss map, and logistic map.

### 3.3. Chaotic Enhanced Genetic Algorithm

In this subsection, the proposed chaotic enhanced genetic algorithm (CEGA) will be described, which is an integration between GA and chaos theory. CEGA be configured so that it uses chaotic noise to overcome any limitations that can be appearing during optimization by GA such as lack of diversity of solutions, the imbalance between exploitation and exploration, and slow convergence of the best solution. CEGA operates in two phases: in the first one, the genetic algorithm is implemented as a global optimization system to solve the NSEs. If the best solution is repeated during the GA optimization process, the chaotic noise is employed as the second phase. Chaotic noise tries to show a sufficient diversity of solutions while preventing time consumption during the optimization process by overcoming the repetition of the best solution and reducing the number of iterations. The following is a full description of the suggested algorithm:

Step 1: initialization
  (i) Individuals of the population (in *n*-dimensions) are created with random placements in the search domain and the number of iterations set to one  (*t*=1)
  (ii) The fitness function *F*(*z*) is assessed for each individual
  (iii) Assign the best individual to the best position ${\left.\hat{\text{Best}}\right)}_{t}$
Step 2: evolution by GA (**t**=**t**+1)
  (i) Ranking [32]: individuals are ranked based on their fitness value, and a vector containing the corresponding individual fitness value is returned, allowing the selection process to compute survival probabilities.
  (ii) Tournament selection (TS) [33]: many solutions (individuals) are chosen at random from the population, and the best of these solutions is chosen to be a parent. This process is performed as many times as necessary to choose parents.
  (iii) BLX-*α* crossover operator [34]: two-parent candidate solutions with *n* design variables, *X*=[*x*_1_, *x*_2_,…, *x*_*n*_] and  *Y*=[*y*_1_, *y*_2_,…, *y*_*n*_], are chosen with crossover probability *P*_*c*_. The BLX-*α* operator creates the *k*-th component of a new offspring *W*. The *k*-th component of *W* is a uniform random scalar in the range [min(*x*_*k*_, *y*_*k*_) − *αI*, max(*x*_*k*_, *y*_*k*_)+*αI*], where *I* defines the distance between parent candidates given by *I*=max(*x*_*k*_, *y*_*k*_) − min(*x*_*k*_, *y*_*k*_) and *a* is a user-defined parameter.
  The BLX-*α* efficacy comes from its capacity to seek in a space domain that is not always constrained by the parents. Furthermore, because the search space is dependent on the distance between the parents, the GA is self-adaptive. The parameter *α* must be chosen carefully since it quantitatively specifies the search domain. Based on the findings of Herrera et al. [35], we choose *α*=0.5 in this investigation.
  (iv) Real-valued mutation [36]: randomly generated values are added to the variables for each new offspring with a low probability (*P*_*m*_) as follows:
    (3) $$\begin{array}{c} Var_{i}^{Mut}=Var_{i}\pm s_{i}\cdot r_{i}\cdot a_{i},\;i\in \left\{1,2,\ldots ,n\right\},\;\text{uniform at random} \end{array}$$
  where *s*_*i*_ ∈ {−1, +1} uniform at random, *r*_*i*_=*r* · *do*  *main*_*i*_, *r*  is mutation range (standard: 10%), *a*_*i*_=2^−*u*·*m*^,  *u* ∈ [0,1] uniform at random, and *m* is mutation precision.
  (v) Elitist strategy: the best individuals in the generation *t* − 1 are directly added to the new generation *t*.
  (vi) Evaluation: for each individual, *F*(*z*) is evaluated to find the new best position ${\left.\hat{\text{Best}}\right)}_{t}$.
  (vii) Updating: if the new best position ${\left.\hat{\text{Best}}\right)}_{t}$ is worse than or equal to the previous best position ${\left.\hat{\text{Best}}\right)}_{t-1}$, go to Step 3. Otherwise, continue by updating the best position as the best individual position discovered so far as ${\left.\hat{\text{Best}}\right)}_{t}.$
  (viii) Termination criteria: the proposed algorithm is terminated when the maximum number of iterations is achieved or when the individual convergences. Convergence happens when the locations of all individuals in the population are identical. Finally, put out the optimal solution as the best individual position ${\left.\hat{\text{Best}}\right)}_{t}$.
Step 3: chaotic noise
  (i) Chaotic noise: chaotic noise is applied if the best solution is repeated during the GA optimization process. It tries to show a sufficient diversity of solutions while preventing time consumption during the optimization process by overcoming the repetition of the best solution and reducing the number of iterations. In this step, the population at generation *t*(POP_*t*_) is changed by chaotic noise as follows:
    (4) $$\begin{array}{c} {\text{POP}}_{t}=\vartheta .{\text{POP}}_{t}, \end{array}$$
  where *ϑ* is a chaotic random number generated by the logistic map by using the following equation:
    (5) $$\begin{array}{c} \vartheta_{q}=4\vartheta_{q-1}\left(1-\vartheta_{q-1}\right),\;\vartheta_{0}\in \left(0,1\right)\;,\;\vartheta_{0}\notin \left\{0.0,0.25,0.50,\;0.75,1.0\right\}. \end{array}$$
  The logistic map, according to the results in [37], improves the quality of the solutions and provides the best performance.
  (ii) Evaluation: for each individual in *POP*_*t*_, *F*(*z*) is evaluated to find the new best position ${\left.\hat{\text{Best}}\right)}_{t}$.
  (iii) Updating: if the new best position ${\left.\hat{\text{Best}}\right)}_{t}$ is better than the previous best position ${\left.\hat{\text{Best}}\right)}_{t-1}$, update the best position ${\left.\hat{\text{Best}}\right)}_{t+1}$ as the best individual's position found so far and continue and go to Step 2. Otherwise, repeat Step 3.

**Figure 2:**
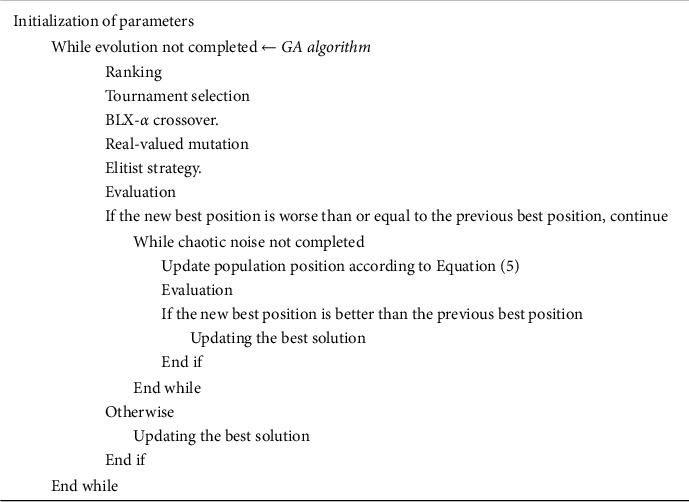
The pseudocode of the proposed algorithm.

Figure 2 depicts the suggested algorithm's pseudocode.

## 4. Numerical Results

Four systems of nonlinear equations are solved to assess the suggested method. These four test systems are common challenges that have been explored by other researchers and are known as benchmarks. The proposed algorithm is coded in MATLAB R2012b and implemented on the PC with Intel(R) Core(TM) i7-6600U CPU @ 2.60 GHz, 16 GB RAM, and Windows 10 operating system. The results will be compared to those obtained by the original GA to demonstrate the benefits of the suggested modifications and their impact on achieving an optimal solution.

For computational studies, a population size equal to 20, generation gap (GGAP) is 0.9, crossover probability *Pc* is 0.8, and mutation probability *P*_*m*_ is 0.02. Also, the termination criterion for CEGA is defined as

(6) $$\begin{array}{c} \delta =\left|\left\|F_{\text{optimum}}\right\|-\left\|F_{t}\right\|\right|\leq \varepsilon =1e-20. \end{array}$$

*F*_*optimum*_ is the optimum value of the objective function which is 0 in all nonlinear system cases while *F*_*t*_ is the calculated objective function at each iteration *t*. It should be noted that the maximum number of iterations for both algorithms (original GA and CEGA) is the same, and all results are recorded from the first run. Furthermore, when one of them meets the termination requirement, the computations stop and the number of used iterations is reported. Finally, to statistically evaluate the CEGA compared to other algorithms, the Friedman test and Wilcoxon rank-sum test are executed here.

### 4.1. Benchmark 1: Experiment Test

This benchmark problem can be described as [7]

(7) $$\begin{array}{c} \left\{\begin{array}{c} f_{1}\left(z_{1},z_{2}\right)=\text{cos}\left(2z_{1}\right)-\text{cos}\left(2z_{2}\right)-0.4=0,\\ f_{2}\left(z_{1},z_{2}\right)=2\left(z_{2}-z_{1}\right)+\text{sin}\left(2z_{2}\right)-\text{sin}\left(2z_{1}\right)-1.2=0,\\ z_{1}\in \left[-10,10\right],\\ z_{2}\in \left[-10,10\right]. \end{array}\right. \end{array}$$

**Figure 3:**
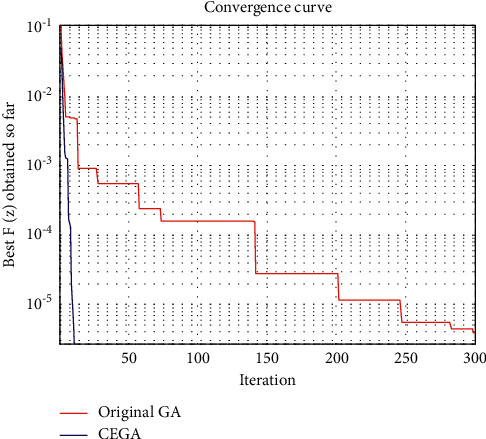
Benchmark 1: the convergence curves of the best *F*(*z*) achieved so far by original GA and CEGA.

This benchmark is solved by many algorithms such as Newton's method, Secant's method, evolutionary algorithm approach (EAA) [7], genetic algorithms (GAs) [21], and hybridization of grasshopper optimization algorithm with genetic algorithm (hybrid-GOA-GA) [38].  Table 1 shows a comparison between the best function value *F* obtained by such algorithms, original GA, and the proposed CEGA. The convergence curves of the best *F*(*z*) achieved so far using original GA and CEGA are shown in Figure 3.

### 4.2. Benchmark 2: Arithmetic Application

This benchmark problem can be described as [7]

(8) $$\begin{array}{c} \left\{\begin{array}{c} f_{1}\left(z\right)=z_{1}-0.254287220-0.18324757\times z_{4}z_{3}z_{9}=0,\\ f_{2}\left(z\right)=z_{2}-0.378421970-0.16275449\times z_{1}z_{10}z_{6}=0,\\ f_{3}\left(z\right)=z_{3}-0.271625770-0.16955071\times z_{1}z_{2}z_{10}=0,\\ f_{4}\left(z\right)=z_{4}-0.198079140-0.15585316\times z_{7}z_{1}z_{6}=0,\\ f_{5}\left(z\right)=z_{5}-0.441667280-0.19950920\times x_{7}x_{6}x_{3}=0,\\ f_{6}\left(z\right)=z_{6}-0.146541130-0.18922793\times z_{8}z_{5}z_{10}=0,\\ f_{7}\left(z\right)=z_{7}-0.429371610-0.21180486\times z_{2}x_{5}x_{8}=0,\\ f_{8}\left(z\right)=z_{8}-0.070564380-0.17081208\times z_{1}z_{7}z_{6}=0,\\ f_{9}\left(z\right)=z_{9}-0.345049060-0.19612740\times z_{10}z_{6}z_{8}=0,\\ f_{10}\left(z\right)=z_{10}-0.426511020-0.21466544\times z_{4}z_{8}z_{1}=0,\\ -10\leq z_{1},\ldots ,z_{10}\leq 10. \end{array}\right. \end{array}$$

**Figure 4:**
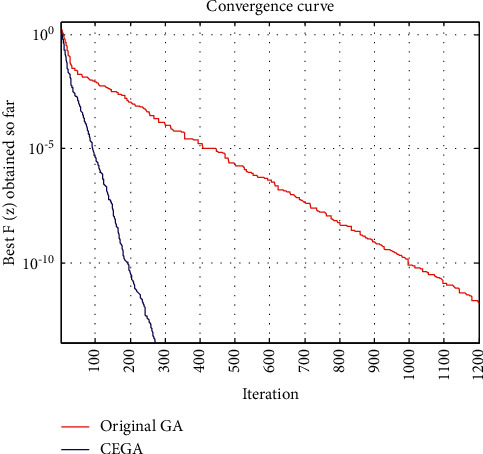
Benchmark 2: the convergence curves of the best *F*(*z*) achieved so far by original GA and CEGA.

This benchmark is solved by many algorithms as the EAA [7], GAs [21], and hybrid-GOA-GA [38].  Table 2 shows a comparison between the best function value *F* obtained by such algorithms, original GA, and the proposed CEGA while the convergence curves of the best *F*(*z*) achieved so far using original GA and CEGA are shown in Figure 4.

### 4.3. Benchmark 3: Combustion Application

This benchmark problem can be described as [7]

(9) $$\begin{array}{c} \left\{\begin{array}{c} f_{1}\left(z\right)=z_{2}+2z_{6}+z_{9}+2z_{10}-10^{-5}=0,\\ f_{2}\left(z\right)=z_{3}+z_{8}-3\times 10^{-5}=0,\\ f_{3}\left(z\right)=z_{1}+z_{3}+2z_{5}+2z_{8}+z_{9}+z_{10}-5\times 10^{-5}=0,\\ f_{4}\left(z\right)=z_{4}+2z_{7}-10^{-5}=0,\\ f_{5}\left(z\right)=0.5140437\times 10^{7}z_{5}-z_{1}^{2}=0,\\ f_{6}\left(z\right)=0.1006932\times 10^{-6}x_{6}-2z_{2}^{2}=0,\\ f_{7}\left(z\right)=0.7816278\times 10^{-15}z_{7}-z_{4}^{2}=0,\\ f_{8}\left(z\right)=0.1496236\times 10^{-6}z_{8}-z_{1}z_{3}=0,\\ f_{9}\left(z\right)=0.6194411\times 10^{-7}z_{9}-z_{1}z_{2}=0,\\ f_{10}\left(z\right)=0.2089296\times 10^{-14}z_{10}-z_{1}z_{2}^{2}=0,\\ -10\leq z_{1},z_{2},\ldots ,z_{10}\leq 10. \end{array}\right. \end{array}$$

**Figure 5:**
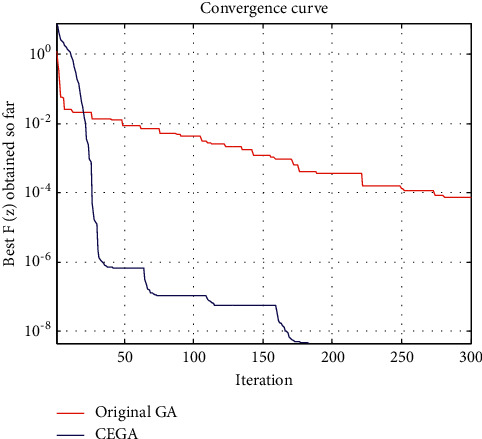
Benchmark 3: the convergence curves of the best *F*(*z*) achieved so far by original GA and CEGA.

This benchmark is solved by many algorithms as the EAA [7], GAs [21], and hybrid-GOA-GA [38].  Table 3 shows a comparison between the best function value *F* obtained by such algorithms, original GA, and the proposed CEGA, while Figure 5 shows the convergence curves of the best *F* obtained so far by original GA and CEGA.

### 4.4. Benchmark 4: Neurophysiology Application

This benchmark problem can be described as [7]

(10) $$\begin{array}{c} \left\{\begin{array}{c} f_{1}=z_{1}^{2}+z_{3}^{2}-1=0,\\ f_{2}=z_{2}^{2}+z_{4}^{2}-1=0,\\ f_{3}=z_{5}z_{3}^{3}+z_{6}z_{4}^{3}=0,\\ f_{4}=z_{5}z_{1}^{3}+z_{6}z_{2}^{3}=0,\\ f_{5}=z_{5}z_{1}z_{3}^{2}+z_{6}z_{4}^{2}z_{2}=0,\\ f_{6}=z_{5}z_{1}^{2}z_{3}+z_{6}z_{2}^{2}z_{4}=0,\\ -10\leq z_{1},z_{2},...,z_{6}\leq 10. \end{array}\right. \end{array}$$

**Figure 6:**
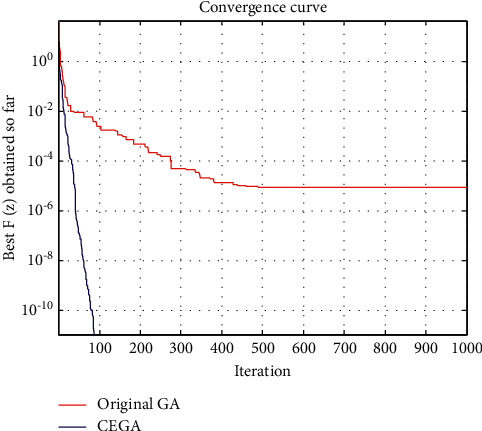
Benchmark 4: the convergence curves of the best *F*(*z*) achieved so far by original GA and CEGA.

This benchmark is solved by many algorithms as the EAA [7], GAs [21], and hybrid-GOA-GA [38].  Table 4 shows a comparison between the best function value *F* obtained by such algorithms, original GA, and the proposed CEGA while Figure 6 shows the convergence curves of the best *F* obtained so far by original GA and CEGA.

## 5. Discussions

Tables 1–4 show the results of all algorithms for the four benchmark problems in terms of the best-obtained solution and the number of iterations. We can observe, for the 1st benchmark problem (experiment test), that hybrid-GOA-GA [38] surpassed the other algorithms in reaching the lowest value of *F*(*z*), which is 1.7904E − 06, but in the number of iterations of 300 while the proposed CEGA obtained a solution very close to the solution obtained by hybrid-GOA-GA, which is 2.7227E − 06, but in only 11 iterations. For the 2^nd^ benchmark problem (arithmetic application), we find that the proposed CEGA outperformed the rest of the algorithms in obtaining the lowest value of *F*(*z*), which is 3.0855E − 14, in 272 iterations, while GAs [21] got an acceptable solution, which is 1.2674E − 09, in the least number of iterations, which is 10. For the 3^rd^ benchmark problem (combustion application), we find that hybrid-GOA-GA [38] outperformed the rest of the algorithms in obtaining the lowest value of *F*(*z*), which is 1.2499E − 09, in 300 iterations. CEGA got an acceptable solution, which is 4.5300E − 09, in an acceptable number of iterations, which is 183, while GAs [21] obtained a reasonable solution, which is 1.8034E − 05, in the fewest number of iterations, which is 70. Finally, for the 4^th^ benchmark problem (Neurophysiology application), we find that the proposed CEGA outperformed the rest of the algorithms in obtaining the lowest value of *F*(*z*), which is 1.0693E − 11, in 87 iterations, while GAs [21] got an acceptable solution, which is 5.2127E − 11, in the least number of iterations, which is 20.

On the other hand, we can see that the original GA' convergence curves had several straight portions, which reflect periods of nonimproving in the objective function owing to entrapment in a local minimum as seen in Figures 3–6, while for CEGA, it is clear that the chaotic noise was successful in permanently improving the objective function and not repeating solutions or spending time on iterations that did not enhance the objective function. The following percentage relationship (IMP%) is used to indicate the improvement between the original GA and the proposed CEGA algorithm:

(11) $$\begin{array}{c} \text{IMP\%}=\frac{\left|\text{original GA iterations}-\text{CEGA iterations}\right|}{\text{original GA iterations}}\times 100. \end{array}$$

As indicated in Table 5, CEGA improved all results significantly by 75.99% on average. So, we can say that chaotic noise guides GA to eliminate the local minimum and enhance the search results, reducing the number of iterations and, as a result, time, by preventing iterations from being used without improvement or convergence to the best solution.

The EAA [7], GAs [21], hybrid-GOA-GA [38], original GA, and the proposed CEGA solved the 4 benchmark problems. Therefore, a statistical evaluation of CEGA compared to these algorithms will be done, according to the best function value *F(z)* by implementing the Friedman test [39] and the Wilcoxon signed-rank test [40] here. The Friedman test compares the algorithms' average ranks and produces Friedman statistics, where the smaller the ranking, the better the performance of the algorithm while the Wilcoxon signed-rank test is used to show the significant differences between the CEGA and the other algorithms.

The Friedman test results are shown in Table 6. Table 6 shows that the Asymp. Sig. (*P* value) is smaller than 0.05, indicating that there are variations in the outcomes obtained by all algorithms. Furthermore, with a lower mean rank, the suggested CEGA algorithm outperforms the other algorithms.

Table 7, on the other hand, displays the results of the Wilcoxon signed-rank test. The sum of positive ranks is R+, whereas the sum of negative ranks equals R−. Table 7 demonstrates that CEGA achieves better R+ values than R− values in 3 cases and is equal in 1 case, indicating that it outperforms other algorithms. As a result of Table 7, we can infer that the proposed CEGA is a significant algorithm and better than the other algorithms.

## 6. Conclusions

In this paper, a chaotic enhanced genetic algorithm (CEGA) to solve the nonlinear system of equations (NSEs) is proposed, which is a combination of genetic algorithm (GA) and chaos theory. CEGA was designed by using a new definition which is chaotic noise to solve the shortcomings of original GA such as a lack of solution variety, an imbalance between exploitation and exploration, repeating best solution throughout the optimization process, and sluggish convergence of the optimal solution. NSEs are first transformed into an unconstrained optimization problem, which is then solved using CEGA.

Four benchmarks problems were considered, which are experiment test, arithmetic application, combustion application, and neurophysiology application. The results obtained by CEGA and the original GA showed that CEGA leads to faster convergence and is successful in finding the optimal solution in fewer iterations than the original GA with an average improvement percentage of about 75.99. On the other hand, the convergence curves showed how the original GA consumes time in trapping into the local minima while the CEGA, by using the chaotic noise, terminated this sticking in the local minimum and moved the optimization process to new better search space. In addition, by comparing CEGA results with other studies, we find that CEGA is competitive and the best. Furthermore, statistical analysis by Friedman and Wilcoxon's tests showed the significance of the CEGA findings, where it got the lowest mean rank and achieved better R^+^ values than R^−^ values.

In our future works, three directions will be concentrated: (i) implementing more modifications for CEGA and assessing their impact on optimization results, (ii) applying CEGA to solve optimization problems in different fields, and (iii) using other metaheuristic algorithms to solve this kind of problems, such as particle swarm optimization [41], ant colony optimization [42], artificial bee colony (ABC) Algorithm [43], krill herd [44], monarch butterfly optimization (MBO) [45], earthworm optimization algorithm (EWA) [46], elephant herding optimization (EHO) [47], moth search (MS) algorithm [48], slime mould algorithm (SMA) [49], hunger games search (HGS) [50], Runge Kutta optimizer (RUN) [51], colony predation algorithm (CPA) [52], and harris hawks optimization (HHO) [53].

## Figures and Tables

**Table 1:** Results for benchmark 1, Experiment test.

Method	(*z*_1_, *z*_2_)	(*f*_1_, *f*_2_)	*F*(*z*)	No. of iterations
Newton's method	(0.15, 0.49)	(0.00168, 0.01497)	0.0083	NA
Secant's method	(0.15, 0.49)	(0.00168, 0.01497)	0.0083	NA
EAA	(0.15722, 49458)	(0.001264, 0.000969)	0.0011	150
GAs	(0.156522, 0.49338)	(4.8606E − 06, 3.7164E − 06)	4.2885E − 06	10
Hybrid-GOA-GA	(0.680235945188233, 2.25999176017399)	(2.2840E − 06, 1.2967E − 06)	**1.7904E** − **06**	300
Original GA	(−2.98506954610277, −2.64821484596259)	(5.2059E − 07, 7.4084E − 06)	3.9645E − 06	300
CEGA	(−9.26825582324219, −8.93140064444864)	(2.9827E − 07, 5.1472E − 06)	2.7227E − 06	**11**

**Table 2:** Results for benchmark 2, Arithmetic application.

Method	*z*_1_⟶*z*_10_		*f*_1_⟶*f*_10_		*F*(*z*)	No. of iterations
EAA	*z*_1_	0.2077500302	*f*_1_	0.0464943	0.2344	300
	*z*_2_	0.0299198492	*f*_2_	0.3489889		
	*z*_3_	−0.0339491324	*f*_3_	0.3058418		
	*z*_4_	−0.2027950317	*f*_4_	0.4012915		
	*z*_5_	0.2131771707	*f*_5_	0.2284027		
	*z*_6_	0.0568458067	*f*_6_	0.0886970		
	*z*_7_	0.2267650517	*f*_7_	0.2024745		
	*z*_8_	−0.0977041236	*f*_8_	0.1687259		
	*z*_9_	−0.0339921200	*f*_9_	0.3787652		
	*z*_10_	0.2532921324	*f*_10_	0.1741025		

GAs	*z*_1_	2.5783339E − 01	*f*_1_	−7.3844E − 10	1.2674E − 09	10
	*z*_2_	3.8109715E − 01	*f*_2_	−1.1684E − 12		
	*z*_3_	2.7874502E − 01	*f*_3_	1.7931E − 09		
	*z*_4_	2.0066896E − 01	*f*_4_	−8.8837E − 10		
	*z*_5_	4.4525142E − 01	*f*_5_	−4.5866E − 10		
	*z*_6_	1.4918391E − 01	*f*_6_	−5.270E − 09		
	*z*_7_	4.3200969E − 01	*f*_7_	−6.3852E − 09		
	*z*_8_	7.3402777E − 02	*f*_8_	−9.7362E − 10		
	*z*_9_	3.4596683E − 01	*f*_9_	−6.0389E − 11		
	*z*_10_	4.2732628E − 01	*f*_10_	3.0841E − 10		

Hybrid-GOA-GA	*z*_1_	0.2578333	*f*_1_	1.2656E − 12	1.7220E − 12	1200
	*z*_2_	0.3810971	*f*_2_	7.9096E − 14		
	*z*_3_	0.2787450	*f*_3_	1.7517E − 12		
	*z*_4_	0.2006689	*f*_4_	4.5315E − 12		
	*z*_5_	0.4452514	*f*_5_	1.1361E − 12		
	*z*_6_	0.1491839	*f*_6_	2.2230E − 12		
	*z*_7_	0.4320096	*f*_7_	1.4795E − 12		
	*z*_8_	0.0734027	*f*_8_	6.5123E − 13		
	*z*_9_	0.3459668	*f*_9_	3.5476E − 12		
	*z*_10_	0.4273262	*f*_10_	5.5468E − 13		

Original GA	*z*_1_	0.257833393700735	*f*_1_	2.6685E − 13	1.7873E − 12	1200
	*z*_2_	0.381097154600942	*f*_2_	1.8415E − 12		
	*z*_3_	0.278745017345425	*f*_3_	1.0000E − 12		
	*z*_4_	0.200668964224041	*f*_4_	1.3058E − 12		
	*z*_5_	0.445251424840196	*f*_5_	8.3411E − 13		
	*z*_6_	0.149183919967650	*f*_6_	1.8859E − 12		
	*z*_7_	0.432009698988807	*f*_7_	4.9226E − 12		
	*z*_8_	0.0734027777813010	*f*_8_	5.0493E − 12		
	*z*_9_	0.345966826875570	*f*_9_	3.8700E − 14		
	*z*_10_	0.427326275994071	*f*_10_	7.2846E − 13		

CEGA	*z*_1_	0.257833393700561	*f*_1_	5.7399E − 14	3.0855E − 14	272
	*z*_2_	0.381097154602820	*f*_2_	1.2136E − 14		
	*z*_3_	0.278745017346455	*f*_3_	1.3031E − 14		
	*z*_4_	0.200668964225329	*f*_4_	1.5905E − 14		
	*z*_5_	0.445251424841115	*f*_5_	7.1657E − 14		
	*z*_6_	0.149183919969369	*f*_6_	1.4279E − 14		
	*z*_7_	0.432009698983808	*f*_7_	8.7737E − 14		
	*z*_8_	0.0734027777762290	*f*_8_	2.1295E − 14		
	*z*_9_	0.345966826875559	*f*_9_	4.9712E − 15		
	*z*_10_	0.427326275993280	*f*_10_	1.0141E − 14		

**Table 3:** Results for benchmark 3, Combustion application.

Method	*z*_1_⟶*z*_10_		*f*_1_⟶*f*_10_		*F*(*z*)	No. of iterations
EAA	*z*_1_	2.8724570E − 4	*f*_1_	−9.0156756E − 5	−1.8038E − 05	300
	*z*_2_	4.6449359E − 004	*f*_2_	−3.3881318E − 021		
	*z*_3_	−3.8722475E − 006	*f*_3_	−5.9848143E − 008		
	*z*_4_	5.7046411E − 005	*f*_4_	−9.0000000E − 005		
	*z*_5_	1.2033492e + 000	*f*_5_	−2.0652682E − 008		
	*z*_6_	3.2144041e + 000	*f*_6_	−1.0783996E − 007		
	*z*_7_	−2.3523205E − 005	*f*_7_	−3.2542930E − 009		
	*z*_8_	3.3872248E − 005	*f*_8_	1.1173545E − 009		
	*z*_9_	1.6152635e + 000	*f*_9_	−3.3367727E − 008		

GAs	*z*_1_	7.7944699E − 5	*f*_1_	−9.0000000E − 5	−1.8034E − 05	70
	*z*_2_	2.3453123E − 4	*f*_2_	−4.7433845E − 20		
	*z*_3_	5.6870072E − 8	*f*_3_	−5.5091023E − 18		
	*z*_4_	−5.1124010E − 4	*f*_4_	−9.0000000E − 5		
	*z*_5_	1.1665683E − 1	*f*_5_	−7.8705351E − 11		
	*z*_6_	3.6717284E − 1	*f*_6_	−7.3037986E − 8		
	*z*_7_	2.6062005E − 4	*f*_7_	−2.6136644E − 7		
	*z*_8_	2.9943130E − 5	*f*_8_	4.7478263E − 14		
	*z*_9_	2.6776713E − 1	*f*_9_	−1.6938693E − 9		
	*z*_10_	−5.0116867E − 1	*f*_10_	−4.2883872E − 12		
	*z*_10_	−4.0222631e + 000	*f*_10_	−6.1982897E − 011		

Hybrid-GOA-GA	*z*_1_	1.5541664E − 9	*f*_1_	8.5611E − 12	1.2499E − 09	300
	*z*_2_	4.6710388E − 6	*f*_2_	1.2440E − 08		
	*z*_3_	2.9852019E − 5	*f*_3_	1.9449E − 14		
	*z*_4_	1.7239638E − 10	*f*_4_	6.6138E − 12		
	*z*_5_	9.8332225E − 6	*f*_5_	5.0547E − 13		
	*z*_6_	2.5029647E − 6	*f*_6_	4.3385E − 11		
	*z*_7_	4.9999104E − 6	*f*_7_	2.5812E − 20		
	*z*_8_	1.3554000E − 7	*f*_8_	2.6115E − 14		
	*z*_9_	9.4779067E − 8	*f*_9_	1.3886E − 15		
	*z*_10_	1.1412198E − 7	*f*_10_	3.3671E − 20		

Original GA	*z*_1_	0.000131595492467185	*f*_1_	1.2576E − 04	7.4518E − 05	300
	*z*_2_	8.25174833157296E − 05	*f*_2_	1.0366E − 04		
	*z*_3_	−2.16100194956660	*f*_3_	1.5119E − 04		
	*z*_4_	−0.00728929937743800	*f*_4_	2.6026E − 05		
	*z*_5_	−2.84721332483602	*f*_5_	1.6368E − 07		
	*z*_6_	−4.25864110800585	*f*_6_	4.4243E − 07		
	*z*_7_	0.00363663681060500	*f*_7_	5.3134E − 05		
	*z*_8_	2.16113561379106	*f*_8_	2.8470E − 04		
	*z*_9_	−1.45063000953809	*f*_9_	1.0072E − 07		
	*z*_10_	4.98385697563882	*f*_10_	8.8564E − 13		

CEGA	*z*_1_	1.15278259019717E − 06	*f*_1_	5.7802E − 11	4.5300E − 09	183
	*z*_2_	9.06471796614326E − 06	*f*_2_	4.4498E − 08		
	*z*_3_	1.56300393104332E − 05	*f*_3_	4.5304E − 10		
	*z*_4_	7.01041293845308E − 06	*f*_4_	4.9701E − 11		
	*z*_5_	2.11248562801178E − 06	*f*_5_	1.2203E − 12		
	*z*_6_	1.28545186382671E − 07	*f*_6_	1.6433E − 10		
	*z*_7_	1.49481838115443E − 06	*f*_7_	4.9146E − 11		
	*z*_8_	1.43254622355700E − 05	*f*_8_	1.5875E − 11		
	*z*_9_	5.33696042558367E − 09	*f*_9_	1.0449E − 11		
	*z*_10_	3.36398449219863E − 07	*f*_10_	9.4722E − 17		

**Table 4:** Results for benchmark 4, Neurophysiology application.

Method	*z*_1_⟶*z*_6_		*f*_1_⟶*f*_6_		*F*(*z*)	No. of iterations
**EAA**	*z*_1_	7.0148122E − 001	*f*_1_	1.1532022E − 009	3.7764E − 10	200
	*z*_2_	7.5925767E − 001	*f*_2_	2.6058267E − 011		
	*z*_3_	−7.1268794E − 001	*f*_3_	−6.5553074E − 010		
	*z*_4_	6.5079013E − 001	*f*_4_	1.1783451E − 009		
	*z*_5_	2.4122542E − 009	*f*_5_	1.1134504E − 009		
	*z*_6_	7.8977724E − 010	*f*_6_	−5.4967453E − 010		

**GAs**	*z*_1_	3.2484137E − 001	*f*_1_	1.5105117E − 010	5.2127E − 11	**20**
	*z*_2_	3.2484137E − 001	*f*_2_	1.5114510E − 010		
	*z*_3_	9.4576852E − 001	*f*_3_	−1.2749912E − 011		
	*z*_4_	9.4576852E − 001	*f*_4_	4.6365863E − 012		
	*z*_5_	−5.6887875E − 001	*f*_5_	1.0181522E − 011		
	*z*_6_	5.6887875E − 001	*f*_6_	8.4981744E − 012		

**Hybrid-GOA-GA**	*z*_1_	0.0820223613267075	*f*_1_	6.9593E − 11	7.0908E − 11	1000
	*z*_2_	−0.138287000903135	*f*_2_	3.1647E − 11		
	*z*_3_	−0.996630489354999	*f*_3_	3.3110E − 12		
	*z*_4_	0.990392197774631	*f*_4_	9.6123E − 12		
	*z*_5_	4.48130330622387E − 09	*f*_5_	2.5478E − 10		
	*z*_6_	4.56992671931472E − 09	*f*_6_	5.6505E − 11		

**Original GA**	*z*_1_	0.00459210535797400	*f*_1_	3.8965E − 11	8.3319E − 06	1000
	*z*_2_	−0.0140392033441710	*f*_2_	8.6758E − 11		
	*z*_3_	0.999989456248088	*f*_3_	3.3069E − 11		
	*z*_4_	−0.999901445571622	*f*_4_	1.3870E − 08		
	*z*_5_	−0.00519291646053100	*f*_5_	4.9063E − 05		
	*z*_6_	−0.00519428784572100	*f*_6_	9.1419E − 07		

**CEGA**	*z*_1_	0.132104801350580	*f*_1_	1.9783E − 11	**1.0693E** − **11**	87
	*z*_2_	0.225320570231597	*f*_2_	1.2026E − 11		
	*z*_3_	−0.991235754742487	*f*_3_	1.6804E − 11		
	*z*_4_	−0.974284681506633	*f*_4_	1.2213E − 12		
	*z*_5_	−1.46708097455544E − 10	*f*_5_	1.0116E − 11		
	*z*_6_	1.36330007947428E − 10	*f*_6_	4.2055E − 12		

**Table 5:** Percentage improvement between the original GA and the proposed CEGA.

Benchmark problem	Original GA	CEGA	IMP% (%)	Average IMP% (%)
Benchmark 1, experiment test	300	11	96.33	75.99
Benchmark 2, arithmetic application	1200	272	77.33	
Benchmark 3, combustion application	300	183	39	
Benchmark 4, neurophysiology application	1000	87	91.3	

**Table 6:** Friedman test.

Ranks		Test statistics	
Method	Mean rank		
EAA [7]	4.50		
GAs [21]	3.25	***N***	4
Hybrid-GOA-GA [38]	1.75	**Chi-square**	11.400
Original GA	4.00	**df**	4
CEGA	**1.50**	**Asymp. Sig. (** *P * **value)**	0.022

**Table 7:** Wilcoxon signed ranks test.

Test statistics		Ranks				
			*N*	Mean rank	Sum of ranks	
**EAA-CEGA**		R^−^	0^a^	0.00	0.00	(a) EAA < CEGA
**Z**	−1.826^m^	**R** ^**+**^	4^b^	2.50	10.00	(b) EAA > CEGA
**Asymp. Sig. (2-tailed)**	0.068	**Ties**	0^c^			(c) EAA = CEGA
m. based on negative ranks	**Total**	4				

**GAs**-**CEGA**		**R** ^**−**^	0^d^	0.00	0.00	(d) GAs < CEGA
**Z**	−1.826^m^	**R** ^**+**^	4^e^	2.50	10.00	(e) GAs > CEGA
**Asymp. Sig. (2-tailed)**	0.068	**Ties**	0^f^			(f) GAs = CEGA
m. based on negative ranks	**Total**	4				

**Hybrid-GOA-GA-CEGA**		**R** ^**−**^	2^g^	3.50	7.00	(g) Hybrid-GOA-GA < CEGA
**Z**	−0.730^n^	**R** ^**+**^	2^h^	1.50	3.00	(h) Hybrid-GOA-GA > CEGA
**Asymp. Sig. (2-tailed)**	0.465	**Ties**	0^i^			(i) Hybrid-GOA-GA = CEGA
n. based on positive ranks	**Total**	4				

**Original GA**-**CEGA**		**R** ^**−**^	0^j^	0.00	0.00	(j) Original GA < CEGA
**Z**	−1.826^m^	**R** ^**+**^	4^k^	2.50	10.00	(k) Original GA > CEGA
**Asymp. Sig. (2-tailed)**	0.068	**Ties**	0^l^			(l) Original GA = CEGA
m. based on negative ranks	**Total**	4				

## Data Availability

All data used to support the findings of this study are included within the article.
